# Monstrosity

**Published:** 1858-03

**Authors:** 


MONSTROSITY. — In the history of double monsters, which was gone over at the time when the Siamese Twins were exhibited, we do not remember if the following description of one was referred to on that occasion. It is given in William of Malmesbury's "Chronicle of the King's of England." *Bohn's Edition*, p. 235.

"At that time (A.D. 1065) on the confines of Brittany and Normandy a prodigy was seen in one, or, more properly speaking, in two women: there were two heads, four arms, and every other part twofold to the navel; beneath were two legs, two feet, and all other parts single. While one was laughing, eating, or speaking, the other would cry, fast, or remain silent; though both mouths ate, yet the excrement was discharged by only one passage. At last one dying, the other survived, and the living carried about the dead for the space of three years, till she died also, through the fatigue of the weight and the stench of the dead carcase."

